# Response of male and female domestic chicks to change in the number (quantity) of imprinting objects

**DOI:** 10.3758/s13420-020-00446-1

**Published:** 2020-10-06

**Authors:** Bastien S. Lemaire, Rosa Rugani, Lucia Regolin, Giorgio Vallortigara

**Affiliations:** 1 Center for Mind/Brain Sciences, University of Trento, Piazza della Manifaturra 1, 38068 Rovereto, TN Italy; 2 Department of General Psychology, University of Padova, Padova, Italy; 3 Department of Psychology, University of Pennsylvania, Philadelphia, PA USA

**Keywords:** Filial imprinting, Domestic chicks, Numerical discrimination, Sex differences

## Abstract

When facing two sets of imprinting objects of different numerousness, domestic chicks prefer to approach the larger one. Given that choice for familiar and novel stimuli in imprinting situations is known to be affected by the sex of the animals, we investigated how male and female domestic chicks divide the time spent in the proximity of a familiar versus an unfamiliar number of objects, and how animals interact (by pecking) with these objects. We confirmed that chicks discriminate among the different numerousnesses, but we also showed that females and males behave differently, depending on the degree of familiarity of the objects. When objects in the testing sets were all familiar, females equally explored both sets and pecked at all objects individually. Males instead selectively approached the familiar numerousness and pecked more at it. When both testing sets comprised familiar as well as novel objects, both males and females approached the larger numerousness of familiar objects. However, chicks directed all their pecks toward the novel object within the set. Differences in the behavior of males and females can be accounted for in terms of sex difference in the motivation to reinstate social contact with the familiar objects and to explore novel ones, likely associated with the ecology and the social structure of the species before domestication.

**Electronic supplementary material:**

The online version of this article (10.3758/s13420-020-00446-1) contains supplementary material, which is available to authorized users.

## Introduction

The investigation of numerical cognition in animals has been challenging. Scientists have been taking advantage of spontaneous choice tasks (where the animals are expected to choose the preferred or the most advantageous option) as well as operant conditioning tasks (Nieder, 2019). Spontaneous preference allows for the investigation of relative numerosity judgments (“more than” or “less than”). Using such procedures, the ability to discriminate between different numerousnesses has been described in several ecological contexts (Nieder, 2020). While foraging, animals from various taxa show a spontaneous preference for more food items (Bogale, Aoyama, & Sugita, 2014; Gazzola, Vallortigara, & Pellitteri-Rosa, 2018; Hauser, Carey, & Hauser, 2000; Hunt, Low, & Burns, 2008; Lucon-Xiccato et al., 2015; Rodríguez et al., 2015; Rugani et al., 2013a, b; Yang & Chiao, 2016). While defending their territory, animals assess the strength of the opponents by estimating their number prior to engaging in defensive displays (Benson-Amram et al., 2011; Bonanni et al., 2011; Cassidy et al., 2015; McComb, Packer & Pusey, 1994; Van Belle & Scarry, 2015; Wilson et al., 2012). While escaping from predators or looking for sexual partners, fishes prefer to join larger groups of conspecifics (Agrillo, Dadda & Bisazza, 2006; Hager & Helfman, 1991; Mehlis et al., 2015; Potrich et al., 2015).

Overall, this evidence shows that animals spontaneously (in the absence of any numerical training) discriminate between numerousnesses to deal with various circumstances in their everyday life. Therefore, animal brains seem to be naturally equipped to use simple numerical cues (Nieder, 2019; Rugani, 2018; Vallortigara, 2015, 2017). Using operant conditioning, several animal species have learned to distinguish stimuli based on the absolute number of items (Bogale et al., 2011; Bortot et al., 2019; Ditz & Nieder, 2016; Pepperberg, 2010; Smirnova, Lazareva, & Zorina, 2000; Xia et al., 2001). By training animals, numerical achievements can reach a high level of abstraction. Beyond numerical discrimination, animals can learn to match symbols to specific numerosities (Biro & Matsuzawa, 2001; Boysen & Berntson, 1989; Olthof, Iden, & Roberts, 1997; Pepperberg & Gordon, 2005), perform simple mathematical operations with those symbols (Boysen & Berntson, 1989; Olthof et al., 1997), symbolically label subsets of items embedded within heterogeneous arrays (Pepperberg & Gordon, 2005) and master the precursors of a zero-like concept (Howard et al., 2018; Merritt, Rugani & Brannon, 2009; Pepperberg, 1988; Pepperberg & Brezinsky, 1991; Pepperberg & Gordon, 2005). Sophisticated and abstract numerical concepts can be mastered by both primates and birds (Pepperberg, 2009; Rugani, Vallortigara, & Regolin, 2016b), indicating that bird brains, though characterized by a different pallial organization (Güntürkün & Bugnyar, 2016), should not be neglected in studying high cognition (Gibbs et al., 2008; Matsushima et al., 2003; Pepperberg, 2017).

To investigate the ontogenetic origins of numerical knowledge, the domestic chick (*Gallus gallus*) is an ideal animal model (Rugani, 2018; Versace & Vallortigara, 2015). Unlike studies on adult animals, chicks can be tested very early in life, allowing for the discovery of the origins of numerical comprehension. Young chicks can learn to solve different numerical problems, ranging from numerical discrimination (Rugani, Vallortigara, & Regolin, 2013b) to the use of ordinal cues (Rugani, Regolin, & Vallortigara, 2007), arithmetic calculation (Rugani et al., 2009), comprehension of proportion (Rugani, Vallortigara, & Regolin, 2015b), and abstract ratios (Rugani et al., 2016a).

Exploiting chicks’ memories for their imprinting objects allows one to assess whether chicks discriminate between different numbers of artificial social companions (i.e., objects they were exposed to soon after hatching; Rugani, Regolin, & Vallortigara, 2008, 2010b). Filial imprinting is a well-known phenomenon (Bolhuis, 1991; McCabe, 2019; Vallortigara & Versace, 2018), allowing the young birds to learn the characteristics of an object they have been exposed to and to develop a robust social attachment toward it in the first few days of life (Bolhuis, 1991; Yamaguchi et al., 2012). Once imprinted, chicks regard their imprinting objects as social companions (Regolin et al., 2005) and can generalize their filial behaviors toward similar objects (Bolhuis & Horn, 1992; Versace et al., 2017). Previous studies have exploited chicks’ memories for their artificial social companions (object they were reared with) to test their numerical abilities, which range from numerical discrimination (Rugani, Regolin, & Vallortigara, 2010b) to proto-arithmetic calculations in the range of small numbers, up to 3 (Rugani et al., 2009), in the large-number range (6 vs. 9 and 5 vs. 10; Rugani, Regolin & Vallortigara, 2011a) and between the small- and large-number range (1 vs. 4 and 2 vs. 5; Rugani et al., 2013a). The upper limit of this kind of numerical discrimination is 3 versus 4. Nevertheless, this limit can be exceeded by the use of cognitive strategies as grouping (Rugani, Loconsole, & Regolin, 2017) and by adding to each object individually distinctive features, allowing for individual processing (Rugani et al., in press).

In 2010, by taking advantage of filial imprinting, it was demonstrated that young domestic chicks, when presented with two sets of objects, prefer to approach the larger one (Rugani et al., 2010b). The choice for familiar or novel stimuli in imprinting situations is known to be affected by the sex of the animals. When exposed to familiar and unfamiliar individuals, males and females of domestic chicks behave differently (Vallortigara & Andrew, 1991; Vallortigara, 1992a, b). Females spend more time close to familiar individuals, whereas males spend more time close to unfamiliar ones. Furthermore, both sexes pecked more at unfamiliar than at familiar individuals, and overall males pecked more than females. These differences are probably the result of different levels of attachment to conspecifics following imprinting (Vallortigara, Cailotto, & Zanforlin, 1990). Females seem to develop stronger attachment to their fellows than males (McBride & Foenander, 1962; McBride, Parer, & Foenander, 1969; Vallortigara et al., 1990; Workman & Andrew, 1989).

The aim of this study was to investigate how male and female chicks respond to familiar and unfamiliar objects in a number (quantity) discrimination test. Considering the different motivation of male and female chicks in approaching their artificial social companion (Regolin et al., 2005), we expected a different behavior depending on the sex of the animals. Using the original method developed by Rugani et al. (2010b), we investigated not only how male and female domestic chicks divided their time in the proximity of a familiar versus an unfamiliar number of objects, but also how they interacted (by social pecking) with familiar and novel objects at test. This new measure allowed us to better understand the factors underlying the intrinsic motivation to join the larger number of artificial social companions.

## Experiment 1

In this experiment, we applied the experimental procedure of the first experiment by Rugani et al. (2010b) to test the discrimination between 1 and 3 imprinting objects in 3-day-old domestic chicks. Here we determined the sex of the animal to assess any difference in the time spent at test by male versus female chicks near the numerically familiar versus numerically novel objects. Moreover, we introduced a novel measure (spontaneous pecking) to assess the level of interaction of each sex based on the scoring of spontaneous pecking toward familiar versus unfamiliar objects at test.

### Ethics statement

The study was performed in compliance with the European Union and the Italian laws on the treatment of animals. The procedures were approved by the Ethics Committee of the University of Trento and licensed by the Italian Health Ministry (permit number 53/2020).

### Subjects

The number of chicks required in each group was a priori determined with a power analysis (Champely, 2020) with an effect size (d) of .65, and an alpha of .05. Results showed that 20 individuals were required per group to achieve a power of .80. Overall, we used 147 chicks (75 females) of the strain Ross 308 (Table 1). The eggs were obtained from a commercial hatchery (Azienda Agricola Crescenti) and were incubated in our laboratory under controlled conditions (37.7 °C and 40% humidity). Three days before hatching, eggs were moved into opaque black boxes within a hatching chamber at 37.7 °C and 60% of humidity.

**Table 1:**
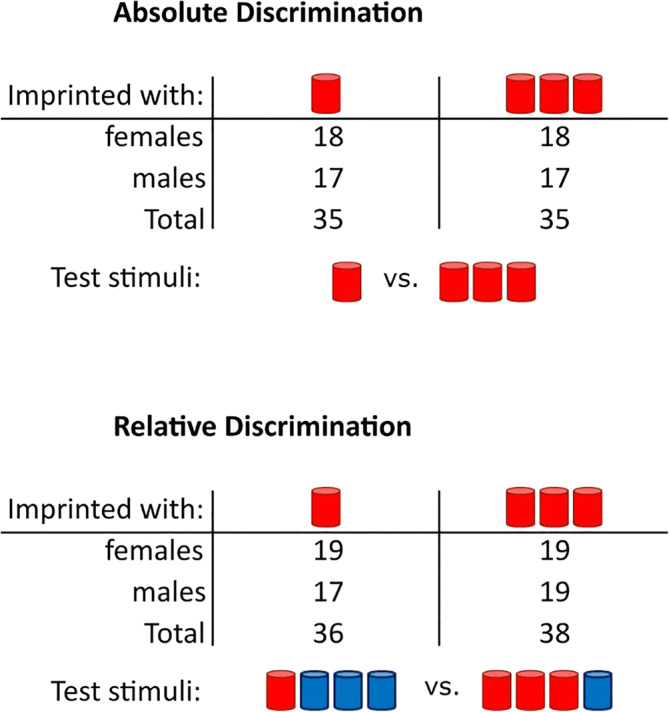
Number of animals and sets of stimuli used in the Absolute and Relative Discrimination tests (chicks that did not respond were excluded from the analysis and therefore are not included in this table). In the Relative Discrimination test, the position of the objects within the sets was fixed

**Fig. 1:**
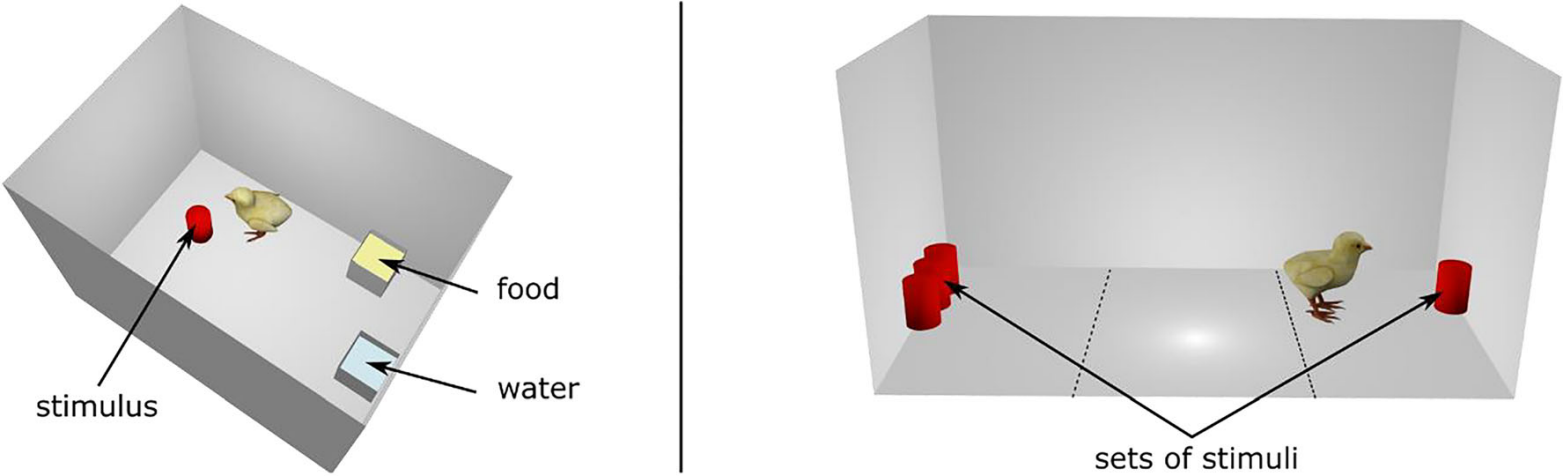
Three-dimensional representation of the rearing cage (**A**) and the testing apparatus (**B**). B shows an example of the *Absolute Discrimination* test. The stimuli were suspended 1 cm above the floor by a transparent thread. Dashed-lines in B delimited the zones (left, center and right) in which the time spent by the animal was scored (the zones were defined computationally using Ethovision)

Soon after hatching, chicks were sexed (this chicken strain exhibits a sexual dimorphism on the wing feathers) and were singly housed into rectangular cages (22 × 30 × 40 cm, Fig. 1A). Cages were illuminated by 30 W fluorescent lights in a controlled temperature environment (30 °C). Chicks were reared together with an artificial stimulus (or set of stimuli) suspended 1 cm above the floor by a transparent thread in the centre of the cage. By exposing the animals to a set of stimuli in their rearing cages, filial imprinting occurred, leading chicks to develop a strong attachment toward those objects. Stimuli consisted of red cylinders (5 cm high, 2 cm diameter per cylinder). Half of the chicks were imprinted to one cylinder, and the other half were imprinted to three cylinders (Table 1). Food (chick starter crumbs) and water were available ad libitum.

After 2 days spent under this rearing conditions (from Tuesday 9:00 am to Thursday 1:00 pm), chicks were individually placed for 6 min in the testing apparatus where they were free to choose and approach either their familiar numerosity set or an unfamiliar numerosity set.

### Test

For testing, animals were moved in a room adjacent to the rearing room and placed into the testing apparatus. This consisted of a short runway (45 × 20 × 30 cm, Fig. 1B), illuminated by two LED lamps (12 V). The behavior of each animal was recorded using a Microsoft Life Camera located 70 cm above the apparatus.

Depending on the experimental condition, two different sets of stimuli were located at either end of the runway (Fig. 1B). Testing stimuli consisted of same size cylinders (5 cm high, 2 cm diameter per cylinder) colored red or blue. Each bird underwent a single test, which could be either an *Absolute Discrimination* or a *Relative Discrimination* test. The position of each set in the runway was balanced across animals (Table 1).

In the *Absolute Discrimination* test, chicks were presented with one versus three objects, which were identical to the imprinting objects (red cylinders, Table 1). In the *Relative Discrimination* task, chicks were presented with two composite sets of four objects. One set comprised one red cylinder (familiar object) and three blue cylinders (unfamiliar objects), while the other set comprised three red cylinders (familiar objects) and one blue cylinder (unfamiliar object, Table 1). The positions of the objects within the sets were fixed (such as presented in Table 1). In the original study, the *Relative Discrimination* test was performed to check whether chicks would respond to the actual number of familiar objects within a set of larger numerosity (composed of unfamiliar objects too).

### Data analysis

To assess the preference toward a set of stimuli, the time spent by the chicks near each set (within a 15-cm area close to it, Fig. 1B) was automatically scored using Ethovision (version 13). A preference for the imprinting numerosity (%) was then calculated using the following formula:

$$ Preference\ for\ imprinting\ numerosity=\frac{time\ spent\ close\ to\ the\ familiar\ imprinting\ numerosity}{time\ spent\ close\ to\ both\ sets\ of\ stimuli}x100. $$

A value higher than 50% indicated a preference for the familiar imprinting numerosity (if imprinted with 1, a preference for 1). A value lower than 50% indicated a preference for the unfamiliar numerosity set (if imprinted with 1, a preference for 3). A score of 50% indicated no preference (chance level).

As pecking is a behavior expressed during social exploration and recognition in domestic chicks (Gottier, 1968; Nicol, 2015; Schjelderup-Ebbe, 1922; Vallortigara, 1992a), we also scored the number of pecks assigned by the animals to each stimulus within each set. Manual scoring was made by a scorer blind to the experimental conditions. The coding reliability of the pecks was assessed by re-coding 21 chicks randomly selected (Pearson’s correlation test showed a high correlation between the two codings, r = 0.99). Pecking analysis was performed once the videorecordings were already archived. Unfortunately, while uploading the videos, a folder got corrupted and prevented us from coding the pecking behaviour of 21 chicks (12 females) in the *Relative Discrimination* test.

Chicks that did not respond (one female and two males) were removed from the analysis as they did not score any preference during the entire testing duration.

### Statistical analysis

To determine whether animals showed preferences for the sets of objects differing across Sex (female, male) and Imprinting Numerosity (1, 3), we performed an ANOVA for each discrimination task (*Absolute*, *Relative*). To meet parametric assumptions, we arcsine-transformed the data. To check whether chicks had a significant preference for the imprinting numerosity, we performed two-tailed one-sample t-tests against chance level (50%).

To determine whether animals were pecking at the stimuli (each cylinder within a set of stimuli) differently across Sex (female, male), we performed an ANOVA for each discrimination task (*Absolute*, *Relative*) and Imprinting Numerosity (1, 3). To meet parametric assumptions, we log-transformed the data.

Post hoc Tukey tests were performed when required using Bonferroni’s correction.

All the statistical analyses were performed using RStudio v1.1 (RStudio Team, 2015) with the following packages: *goftest* (Faraway et al., 2019), *nlme* (Pinheiro et al., 2020), *lme* (Bates et al., 2015), *tidyr* (Wickham & Lionel, 2020), *plyr* (Wickham, 2011), *dplyr* (Wickham et al., 2020), *reshape* (Wickham, 2007), *lsr* (Navarro, 2015)*, ggplot2* (Wickham, 2016), pwr (Champely, 2020).

### Results

#### Absolute Discrimination

##### Preference for the imprinting numerosity

**Fig. 2:**
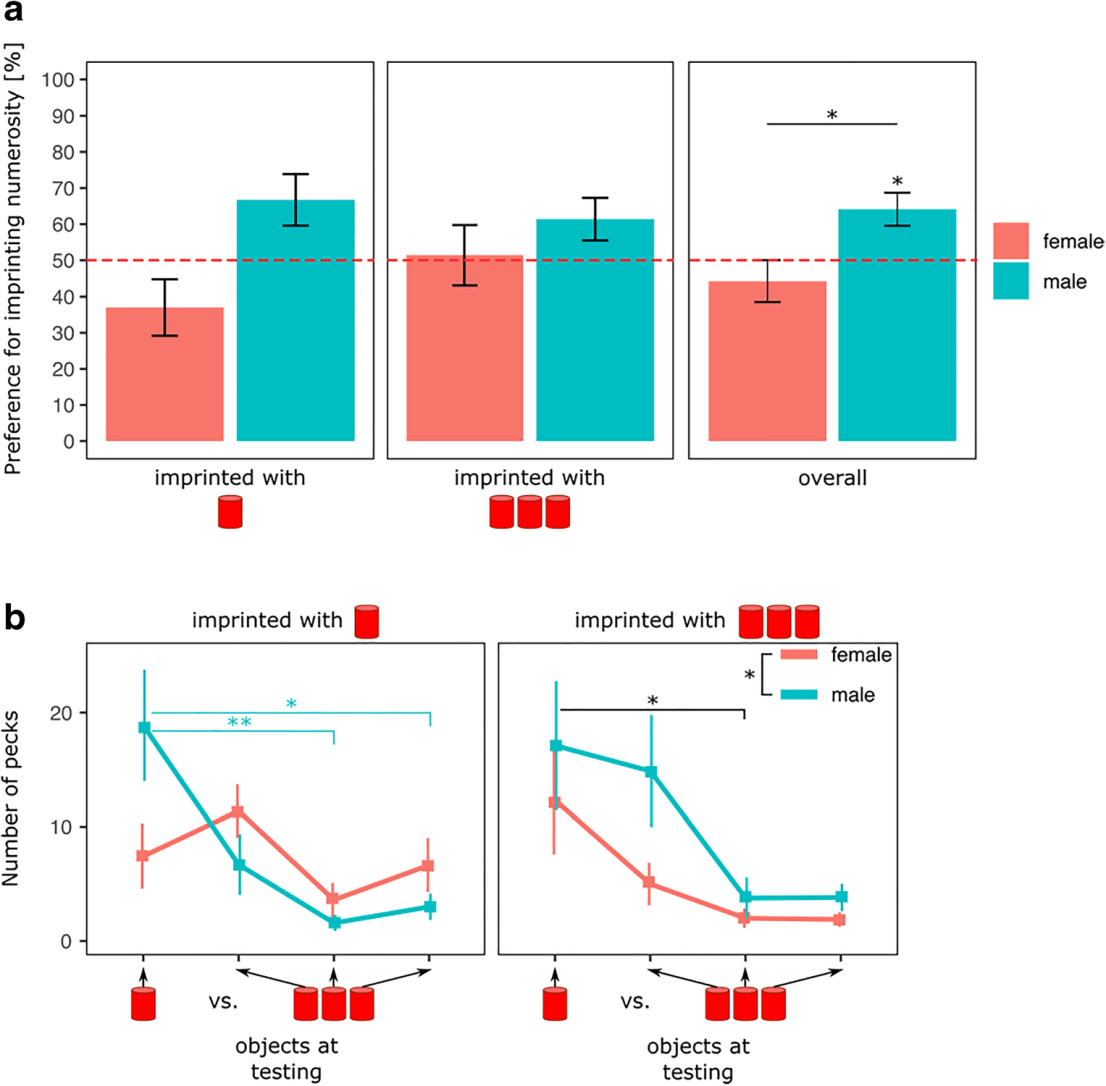
Graph (**A**) represents the time spent by the chicks during testing close to their imprinting numerosity between Imprinting Numerosity and Sex (p < 0.05, *). Graph (**B**) represents the number of pecks made toward each stimulus composing the different set of stimuli between Sex (females in red and males in light-blue) and Imprinting Numerosity (* p < 0.05; ** p < 0.01). Light blue asterisks show the statistical difference in males; black asterisks show the statistical differences in both males and females

The results are shown in Fig. 2A. There was a significant effect of Sex (*F*(1, 66) = 6.73, *p* < 0.05), but not of Imprinting Numerosity (*F*(1, 66) = 0.20, *p* = 0.66) or interaction (*F*(1, 66) = 1.69, *p* = 0.20).

Overall, the preference for the imprinting numerosity set was significantly different from chance level for males (t(33) = 2.50, p < 0.05, Cohen’s d = 0.42, Bonferroni correction), but not for females (t(35) = -0.54, p = 0.59, Cohen’s d = 0.090, Bonferroni correction). Males spent on average 66% (± 5.89 SEM) of their time close to the imprinting numerosity set, while females spent on average 47% (± 6.42 SEM) of their time close to it.

##### Pecks

The results are shown in Fig. 2B. When imprinted with 1, there was a significant effect of Object (F(3, 132) = 6.46, p < 0.01) and of the interaction between Object and Sex (F(3, 132) = 6.36, p < 0.01) but a non-significant effect of Sex (F(1, 132) = 0.86, p = 0.36). While females pecked similarly at each object, the post hoc analysis revealed that males pecked significantly more at the single object in comparison to the central (t(132) = 4.10, p < 0.01, Cohen’s d = 1.41) and right-most (t(132) = 3.50, p < 0.05, Cohen’s d = 1.19) objects within the set of three.

When imprinted with 3, there was a significant effect of Sex (*F*(1, 132) = 4.12, *p* < 0.05) and Object (*F*(3, 132) = 3.99, *p* < 0.01), but no interaction (*F*(3, 132) = 0.30, *p* = 0.82). As revealed by the ANOVA, males (mean = 9.90, SEM = 2.27) pecked significantly more than females (mean = 5.31, SEM = 1.25) at the objects, a difference that seems to mainly be driven by the number of pecks made at the left-most object within the set of three. Males pecked on average 15 times (SEM = 4.79) at it, while females pecked on average five times (SEM = 1.76) at it.

Overall, the post hoc analysis revealed that chicks pecked significantly more at the single stimulus in comparison to the central object composing the set of three stimuli (*t*(132) = 3.05, *p* < 0.05, Cohen’s *d* = 0.73).

### Relative Discrimination

#### Preference for the imprinting numerosity

**Fig. 3:**
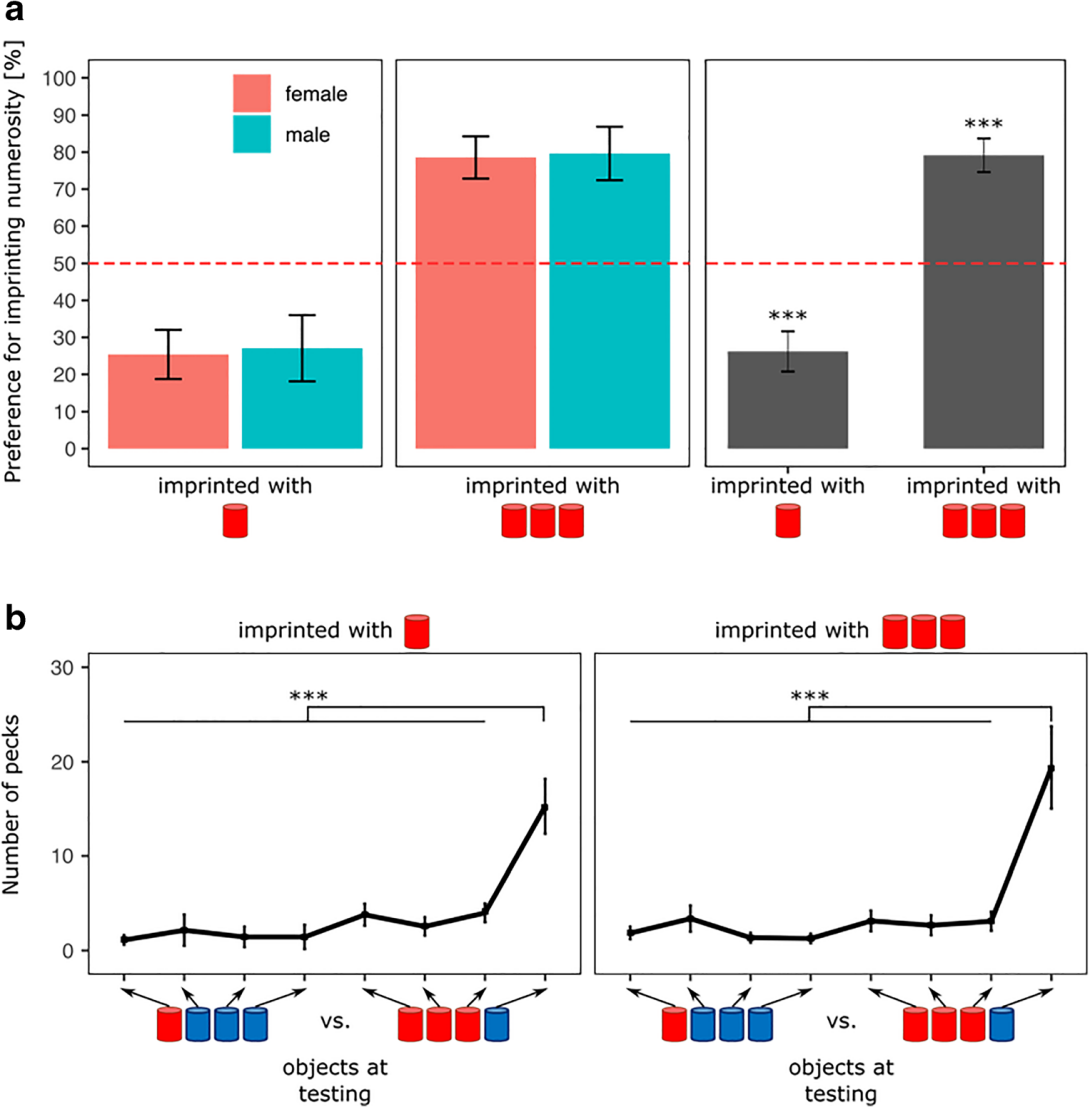
(**A**) The time spent by the chicks during testing close to the object set containing the number of familiar objects experienced during imprinting between Imprinting Numerosity and Sex (p < 0.001, ***). (**B**) The number of pecks made toward each stimulus composing the different set of stimuli between Imprinting Numerosity (females and males grouped; *** p < 0.001). Black asterisks show the statistical differences in both males and females

The results are shown in Fig. 3A. There was a significant effect of Imprinting Numerosity (*F*(1, 70) = 53.16, *p* < 0.001) but no significant effect of Sex (*F*(1, 70) = 0.19, *p* = 0.67) nor of the interaction (*F*(1, 70) = 0.00, *p* = 0.99).

Overall, the preference for the imprinting numerosity set was significantly different from chance level for the chicks imprinted with 1 (*t*(35) = -4.74, *p* < 0.001, Cohen’s d = 0.79, Bonferroni correction) and 3 (t(37) = 5.32, p < 0.001, Cohen’s d = 0.86, Bonferroni correction). Nevertheless, the preferences between these two groups were the opposite. Chicks imprinted with 1 spent on average 23% (±5.64 SEM) of their time close to the imprinting numerosity set while chicks imprinted with 3 spent on average 79% (±5.36 SEM) close to it. This indicates that, independently of rearing conditions and sex, chicks approached the larger set of familiar objects.

#### Pecks

The results are shown in Fig. 3B. When imprinted on 1 or 3, there was a significant effect of Object (imprinted with 1: *F*(7, 184) = 11.48, *p* < 0.001; imprinted with 3: *F*(7, 208) = 7.75, *p* < 0.001), but not a significant effect of Sex (imprinted with 1: *F*(1, 184) = 0.01, *p* = 0.92; imprinted with 3: *F*(1, 208) = 1.10, *p* = 0.30) or of the interaction (imprinted with 1: *F*(7, 184) = 0.36, *p* = 0.93; imprinted with 3: *F*(7, 208) = 0.71, *p* = 0.66).

No matter whether they had been imprinted on 1 or 3, the post hoc analysis revealed that chicks pecked significantly more at the single blue object in the set in comparison to all other objects (statistics are detailed in the Online Supplemental Material table).

## Experiment 2

In the first experiment, chicks behaved differently in the two discrimination tests. When only exposed to familiar objects at test (*Absolute Discrimination*), males approached the familiar numerousness, whereas females did not show any preference. This may indicate a different motivation of male and female birds in exploring a novel numerousness of familiar objects. Males seemed more inclined to spend time close to the familiar numerousness, while females seemed equally motivated in joining the familiar group or exploring the novel one. When tested in the presence of familiar and unfamiliar objects (*Relative Discrimination*), both sexes used the relative numerical information available concerning the subset of familiar objects present within each set and chose to associate with the larger set of familiar objects as described in the original study by Rugani et al. (2010a, b). The presence of novel objects seems to play a relevant role in the choice to approach the larger number of familiar objects, or in avoiding the larger number of unfamiliar ones. Support for the latter hypothesis comes from the analyses of the pecks, which consistently highlight an increased number of pecks at the single novel object in the group comprising three familiar objects and an unfamiliar one.

To investigate the importance of unfamiliarity, we conducted a second experiment in which we manipulated the degree of unfamiliarity.

### Methods

The general procedure was the same as in the first experiment. The same number of objects as in the *Absolute Discrimination* was used (Table 2), but this time we slightly modified the appearance of the stimuli during testing. Chicks at test were offered a choice between one or three objects, but a small yellow dot (diameter of 5 mm) was added on the central object of the set corresponding to the original imprinting numerosity (Table 2). For the chicks that had been imprinted to one object, the yellow dot was placed on the single object during testing. For the chicks that had been imprinted to three objects, the yellow dot was placed on the central object composing the set of three during testing.

**Table 2:**
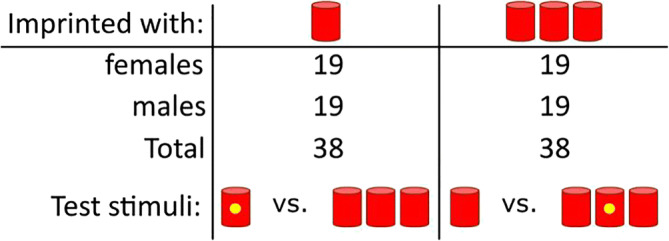
Number of animals and sets of stimuli used in the second experiment (chicks that did not respond were excluded from the analysis and therefore are not included in this table)

We used 79 chicks (40 females) of the strain Ross 308 (Table 2). Chicks that did not respond (two females and one male) were removed from the analysis as they did not score any preference during the entire testing duration.

### Results

#### Preference for the imprinting numerosity

**Fig. 4:**
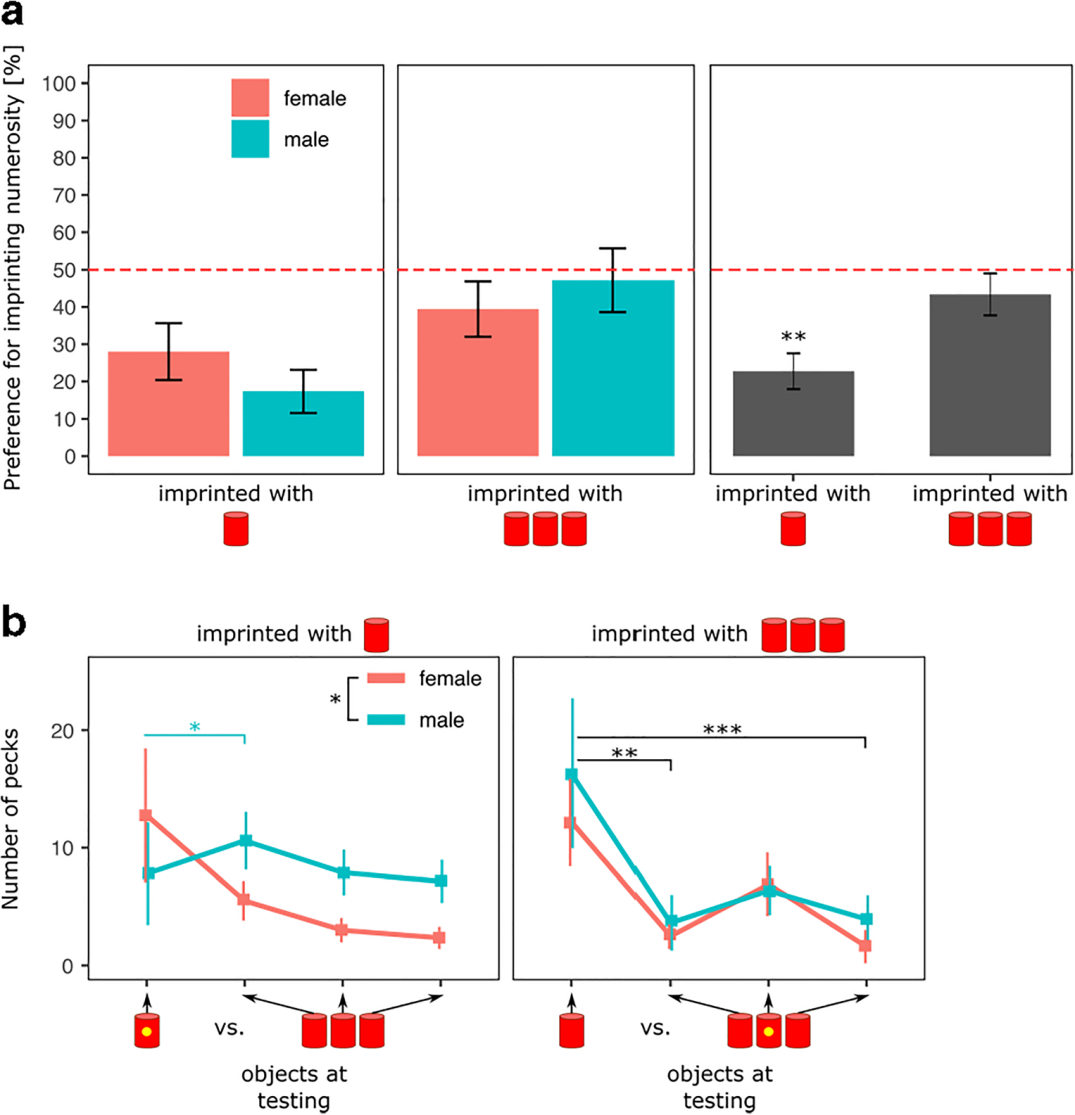
Graph (**A**) represents the time spent by the chicks during testing close to their imprinting numerosity set between Imprinting Numerosity and Sex (*** p < 0.015). Graph (**B**) represents the number of pecks assigned to each stimulus composing the different sets of stimuli between Sex (females in red and males in light-blue) and Imprinting Numerosity (* p < 0.05; ** p < 0.01; *** p < 0.001). Light blue asterisk shows the statistical difference in males; black asterisks show the statistical differences for both males and females

The results are shown in Fig. 4A. There was a significant effect of Imprinting Numerosity (*F*(1, 72) = 7.43, *p* < 0.01) but no significant effects of Sex (*F*(1, 72) = 0.075, *p* = 0.79) or of the interaction (*F*(1, 72) = 1.87, *p* = 0.18).

Overall, the preference for the imprinting numerosity was significantly different from chance level for the chicks imprinted to 1 (*t*(37) = -5.86, *p* < 0.001, Cohen’s *d* = 0.97, Bonferroni correction) but not for the chicks imprinted to 3 (*t*(37) = -0.83, *p* = 0.41, Cohen’s *d* = 0.14, Bonferroni correction). Chicks imprinted to 1 spent on average 20% (±4.98 SEM) of their time close to the familiar numerosity, while chicks imprinted to 3 spent on average 44% (±6.62 SEM) close to it.

#### Pecks

The results are shown in Fig. 4B. When imprinted with 1, there was a significant effect of Sex (*F*(1, 144) = 5.51, *p* < 0.05) and an interaction between Object and Sex (*F*(3, 144) = 4.23, *p* < 0.01), but no significant effect of Object (*F*(3, 144) = 1.96, *p* = 0.12). As revealed by the ANOVA, males were pecking (mean = 8.80, SEM = 1.40) significantly more than females (mean = 6.21, SEM = 1.39) at the objects.

Furthermore, the post hoc analysis revealed that males pecked significantly more at the left-most object (mean = 11.16, SEM = 2.39) in the set of three objects in comparison to the single object (mean = 8.21, SEM = 4.67; *t*(144) = -3.45, *p* < 0.05, Cohen’s *d* = -1.12). The opposite tendency was observed in females although not revealed by the post hoc analysis as the variability of the pecks to the single object seemed quite high (mean = 13.42, SEM = 5.84) in comparison to that of the left-most object within the set of three objects (mean = 5.79, SEM = 1.61).

When imprinted with 3, there was a significant effect of Object (*F*(3, 144) = 6.92, *p* < 0.001) but no significant effects of Sex (*F*(1, 144) = 0.46, *p* = 0.50) nor of the interaction (*F*(3, 144) = 0.23, *p* = 0.87).

The post hoc analysis revealed that chicks pecked significantly more at the single stimulus in comparison to the left-most one (*t*(144) = 3.48, *p* < 0.01, Cohen’s *d* = 0.80) or to the right-most one (*t*(144) = 4.10, *p* < 0.001, Cohen’s *d* = 0.94) in the set of three objects .

## General discussion

Number cognition in birds has been widely investigated. Studies performed by Pepperberg on Alex the parrot illustrated the impressive numerical competences owned by avian species (Pepperberg, 2009). Alex was able to quantify up to six-item sets using English labels with an accuracy of 80%, and remaining unaffected by array quantity, mass, or contour (Pepperberg, 1994; Pepperberg & Gordon, 2005). The achievement of a high level of abstraction in numerical comprehension suggests that birds may naturally deal with numerical information in everyday life. In our study, instead of focusing on the symbolic use of numbers as Pepperberg did, we studied a much simpler numerical ability (numerousness discrimination), exploiting a paradigm that allows one to test numerical comprehension in the absence of any numerical training. This allows for the understanding of how birds can spontaneously deal with numerical cues.

As in previous studies (Rugani et al., 2008, 2010b), our results showed that chicks could use numerical/quantity information to discriminate between different sets of objects. As we did not control for the role of continuous physical variables, we cannot disentangle whether chicks used numerical and/or continuous quantities to discriminate. However, previous work has shown that chicks, depending on the available cues, could use both sources of information (Rugani et al., 2010b). Moreover, several sources of supporting evidence points to the existence of a general magnitude system in human and non-human species that comprises both discrete (countable) and continue quantities (Bortot, Stancher & Vallortigara, 2020; De Corte, Navarro & Wasserman, 2017; Di Giorgio et al., 2019; Gallistel, 1989; Merritt, Casasanto, & Brannon, 2010; Walsh, 2003).

In our study, we focused on whether males and females used numerical/quantities cues similarly. We found that males and females make different use of numerical cues depending on the context (familiar, in the *Absolute Discrimination* task, or familiar and unfamiliar in the *Relative Discrimination* task).

The *Absolute Discrimination* task showed that males prefer to associate with the familiar numerosity set, while females showed no preference. This may indicate that in a novel environment, as the testing apparatus was, males are motivated to approach the familiar numerosity sets, while females are equally motivated to explore the two sets. The *Relative Discrimination* task revealed a completely different pattern of results, but this time more similar to what Rugani et al. (2010b) described initially. Males and females behaved alike and had a strong preference to associate with the set composed of more familiar objects (three red objects and one blue object). Motivational differences may support chicks’ tendency to approach the larger group of social companions: the larger group can guarantee more protection toward potential predators, higher level of social interaction, a richer environment, and in the natural situation more heat (Pulliam, 1973; Roberts, 1996). Furthermore, the experimental procedure used in our study exploits the chick’s memory for its imprinting object (or set of objects). As a result of filial imprinting, an increase in fear is observed when exposed to novel stimuli (Bolhuis, 1991). This leads the young animals to avoid proximity with novel objects, which therefore could explain why chicks tend to associate with the set composed of the largest number of familiar objects and the fewest number of unfamiliar objects.

The number of pecks here is not to be interpreted as a feeding behavior. Instead, it likely reflects a social, either affiliative or aggressive, behavior toward familiar or unfamiliar objects (Vallortigara, 1992a), providing interesting cues as to the possible nature of sex differences. While affiliative pecks are usually equally distributed across time among familiar individuals, aggressive pecks are reiterated toward unfamiliar individuals/objects (Schjelderup-Ebbe, 1935). Pecking behavior may, therefore, reveal lack of recognition when intensively exhibited toward an individual (Guhl & Ortman, 1953; Vallortigara, 1992a, b). In the *Absolute Discrimination* task, males pecked more at the familiar numerosity set. This measure correlates with the time spent near the familiar numerousness and shows a preference of male chicks to interact more with the familiar set rather than with the novel one. A peculiar behavior emerged from the analysis of the distribution of pecks toward the array of three objects: males seemed to peck more at the left object than at the central or at the right one. This left-sided preference could be related to a general bias in the allocation of spatial attention (Diekamp et al., 2005; Regolin, 2006; Rugani et al., 2011b). Day-old domestic chicks, in fact, associate numbers with space in different contexts. Chicks trained to respond to a certain numerical value spontaneously associated a smaller number with the left side and a larger number with the right side of space (Rugani et al., 2020, 2015a). Chicks, trained to identify a target element (e.g., the fourth) in a sagitally oriented series of identical elements, when required to react to an identical series but rotated by 90°, identified most often the left target than the right one (Rugani et al., 2010a, 2015a). A lateral bias has also been found in a numerical task which required to discriminate between two groups of artificial social companions. Female chicks were reared with a set of identical objects. At test, the objects disappeared one at a time behind one of two identical screens, one on the left and one on the right. On a free-choice test, chicks showed a preference for the larger group. Nevertheless, their performance was higher when the larger group was hidden on the right side (Rugani, Rosa Salva, & Regolin, 2014). This evidence suggests that also in a spontaneous search for social companions a tendency can emerge to associate numbers and space. The latter evidence is also in line with our new findings in which male chicks tend to explore the smaller numbers of social companions on the left side. Going back to our current study, females, in contrast, visited and pecked at all objects individually, which can probably explain why they did not express any preference in the time spent analysis (females were at chance-level). Curiously, when imprinted with the set composed of three objects, both sexes significantly pecked more at the single stimulus as if they were treating it as a less familiar individual. Indeed, pecking can also demonstrate a lack of recognition when it is repeatedly directed toward a specific individual (Guhl & Ortman, 1953; Vallortigara, 1992a, b). Hence, it is possible that chicks used numerical/quantitative information available to determine which object was more likely to be unfamiliar.

A similar interpretation can be made by looking at the results obtained in the *Relative Discrimination* task. Chicks repeatedly pecked at the blue object incorporating the larger set of familiar objects (three red objects), demonstrating that they recognized the blue object as being unfamiliar within a familiar set.

Our results suggest that male and female domestic chicks use numerical/quantity information slightly differently depending on the familiarity of the objects. In a familiar context, males tend to use numerical/quantity information to discriminate between two sets of familiar objects, while females focus on each familiar object individually. Interestingly, in a context composed of unfamiliar objects, males and females expressed similar behaviors and chose to associate with the set containing less unfamiliar objects. In the previous study (Rugani et al., 2010b), no difference was found between the *Absolute* and *Relative Discrimination* tasks. Therefore, the authors concluded that chicks mainly use the number of familiar objects to discriminate between two sets of stimuli and that they choose to associate with the larger set of familiar objects. Our data instead support the idea that chicks mainly rely on the number of unfamiliar objects instead of the number of familiar ones. This difference could be possibly explained by the colors used: yellow and light pink in the study by Rugani et al. (2010b), versus red and blue in the present study. The relevance of the unfamiliar object may have been emphasized using novel blue objects instead of light pink ones. Even if different strategies seem to be at the basis of the current study (avoid the larger number of unfamiliar objects) and in the previous study (approach the larger number of familiar objects), nevertheless both studies converge in demonstrating that day-old chicks do discriminate numerousness in the absence of any numerical training. Differences in strains should also be considered.

To better understand to which degree unfamiliarity influences chicks’ behaviors, we conducted a second experiment. The task in this second experiment shared properties with the *Absolute* and *Relative Discrimination* tasks. During testing, chicks were offered a choice between a set of one or three objects (such as in the *Absolute* condition). However, we slightly changed the appearance of the familiar numerosity set so that it neither appeared completely familiar nor completely unfamiliar, by adding a small yellow dot on one of the objects of the familiar numerosity set (for the discriminability of imprinting object based on individual features depicted on them, see Fontanari et al., 2011).

In the second experiment, chicks tended to explore the unfamiliar numerosity set. This demonstrates that even a slight change in the appearance of one object influences the chick’s decision to associate with either set. Although no sex difference was observed in terms of time spent close to either set, the pecking analysis revealed that males and females behaved differently. When imprinted with one, females pecked more at the single object (with the yellow dot) than at the other objects (set of three, where they spent 72% of their time). As in the *Absolute Discrimination* task, females seem to focus more on individual recognition, which suggests that they do not treat the single stimulus (with the yellow dot) as entirely unfamiliar. In contrast, males completely avoided the single stimulus (with the yellow dot) and pecked more at the set of three. The results of this second experiment appear to be in agreement with the study by Rugani, Regolin and Vallortigara (2010b) and with our first experiment, suggesting that chicks firstly discriminate between familiar and unfamiliar objects before making a decision based on their numerousness.

Our study provides additional information concerning the use of numerical or quantitative information by young domestic chicks in the specific context of filial imprinting. Taken together, our results confirm that chicks can use numerousness to discriminate between different set of objects. It also demonstrates that male and female domestic chicks do not always use available numerical cues similarly, and that, instead, they might prefer to use different strategies depending on the familiarity of the objects. Overall, females seem more flexible in the use of numerical/quantitative cues depending on the context. In a familiar context, females perform individual recognition rather than using numerical/quantitative information to make a decision. In an environment composed of familiar as well as unfamiliar objects, females used numerical information to discriminate between two sets of objects. In contrast, males tend to rely upon numerical information either by approaching the familiar numerosity set when exposed to familiar objects or the larger set of familiar objects when exposed to sets of familiar and unfamiliar objects.

Likely the sex difference we observed may derive from the natural history of feral birds. Adult fowls are organized in groups comprising a dominant rooster and many hens (Queiroz & Cromberg, 2006). Males are more solitary as they spend most of their time maintaining and patrolling their territory, whereas females tend to live in strict hierarchies that they develop and maintain through time (Gottier, 1968; McBride & Foenander, 1962; McBride et al., 1969; Schjelderup-Ebbe, 1922). Such organization may favor the prevalence of gregarious and affiliative behaviors in females (Cailotto, Vallortigara, & Zanforlin, 1989; Vallortigara et al., 1990; Vallortigara, 1992b; Workman & Andrew, 1989), as well as greater use of specific abilities such as transitive inference (Daisley, Vallortigara, & Regolin, 2010) favoring individual recognition. Given their roles of chaperone, males might overlook individual recognition and instead rely on cues helping them to assess potential threats promptly. In such a context, using numerical abilities would be an efficient strategy, as has been shown to occur in several other species (Benson-Amram et al., 2011; Bonanni et al., 2011; Cassidy et al., 2015; McComb, Packer, & Pusey, 1994; Van Belle & Scarry, 2015; Wilson et al., 2012).

In conclusion, our results show that young and almost naïve domestic chicks rely on numerical information to make social decisions. They first discriminate between familiar versus unfamiliar objects based on their perceptual features, and then they estimate the numerousness of both sets to avoid the larger number of unfamiliar objects. Moreover, the degree of novelty of the unfamiliar objects seems to correlate with the avoidance of the unfamiliar set. Numerousness is, therefore, a relevant information animals can spontaneously use in a social context to optimize their fitness.

## Electronic supplementary material

ESM 1 (PNG 278 kb)

## Data Availability

The datasets (.csv) are available on Fig **Share** (10.6084/m9.figshare.12336140).
