# Supplementary figures and images for: Response of male and female domestic chicks to change in the number (quantity) of imprinting objects
Source: Learn Behav. 2020 Oct 6;49(1):54–66. doi: 10.3758/s13420-020-00446-1 (PMC7979580; doi:10.3758/s13420-020-00446-1)

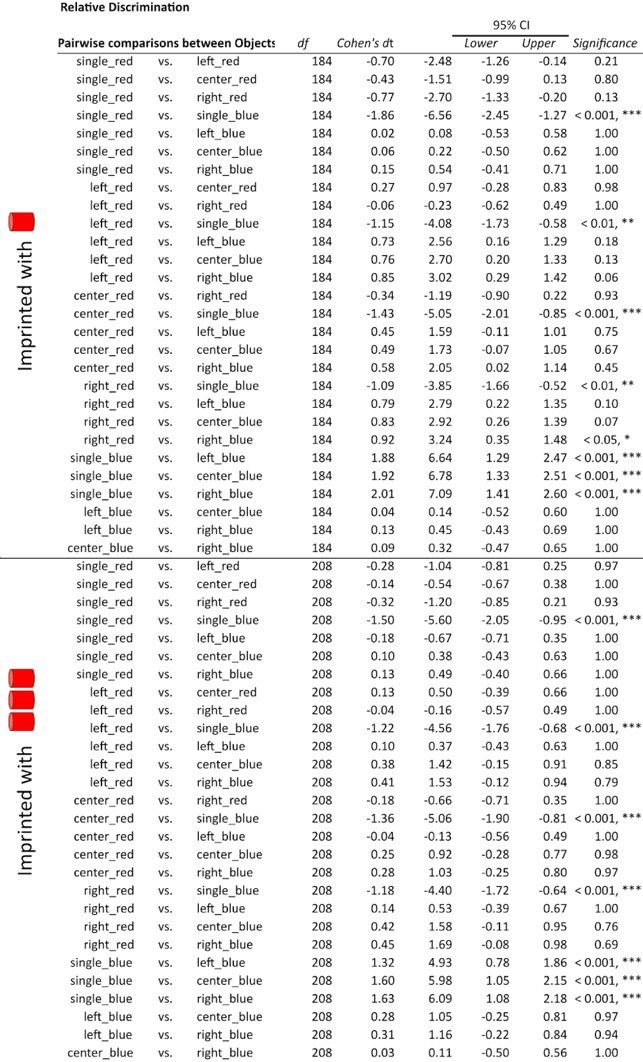

Supplement: Supplementary file 1 — (PNG 278 kb) [file 13420_2020_446_MOESM1_ESM.png]
